# Challenges in Geriatric Oncology—A Surgeon’s Perspective

**DOI:** 10.3390/curroncol29020058

**Published:** 2022-01-29

**Authors:** Ruth Parks, Kwok-Leung Cheung

**Affiliations:** 1 Nottingham Breast Cancer Research Centre, University of Nottingham, Nottingham NG7 2RD, UK; ruth.parks@nottingham.ac.uk; 2 School of Medicine, Royal Derby Hospital Centre, Uttoxeter Road, University of Nottingham, Derby DE22 3DT, UK

**Keywords:** surgical oncologist, geriatrician, older adults, cancer, clinical trials, frailty

## Abstract

As our global population ages, we will see more cancer diagnoses in older adults. Surgery is an important treatment modality for solid tumours, forming the majority of all cancers. However, the management of older adults with cancer can be more complex compared to their younger counterparts. This narrative review will outline the current challenges facing older adults with cancer and potential solutions. The challenges facing older adults with cancer are complex and include lack of high-level clinical trials targeting older adults and selection of the right patient for surgery. This may be standard surgical treatment, minimally invasive surgery or alternative therapies (no surgery) which can be local or systemic. The next challenge is to identify the individual patient’s vulnerabilities to allow them to be maximally optimised for treatment. Prehabilitation has been shown to be of benefit in some cancer settings but uniform guidance across all surgical specialties is required. Greater awareness of geriatric conditions amongst surgical oncologists and integration of geriatric assessment into a surgical clinic are potential solutions. Enhanced recovery programmes tailored to older adults could reduce postoperative functional decline. Ultimately, the greatest challenge an older adult with cancer may face is the mindset of their treating clinicians—a shared care approach between surgical oncologists and geriatricians is required.

## 1. Background

Cancer is a disease of older people, with the incidence of the majority of cancers increasing with age [1]; the highest rates of cancer cases in the UK population in 2015–2017 was in the age group 85–89 years [2]. Surgery is an important treatment modality in the majority of cancers with the exception of haematological malignancies. The role of surgery is vast; it can be utilised for prophylaxis, primary treatment, after neoadjuvant systemic therapy, as well as in the palliation of symptoms and improvement of quality of life in the metastatic setting. Consequently, the average age of the population served by a surgeon is significantly increasing.

In this article we will discuss the main issues facing surgical oncologists and geriatricians at the present time including the differing evidence base upon which to make treatment decisions in older compared to younger adults, as well as how to select the right patient for the most appropriate treatment. We will then discuss the concept of patient optimisation, where surgical treatment is deemed the most appropriate option and finally, focus on developing the relationship between the surgical oncologist and the geriatrician. This article is not intended to give a systematic review of the literature on each subject but rather a comprehensive overview of the topic as a whole.

## 2. Challenges and Potential Solutions

A summary of the challenges and potential solutions facing clinicians treating older adults with cancer is given in Table 1.

### 2.1. Differing Evidence Base for Surgery in Older Adults Compared to Younger

Surgery for older patients with cardiac, orthopaedic or vascular diseases is increasingly accepted; however, the reluctance to offer optimal cancer surgery in this age group continues [3,4], despite recognition in the last decade that this is an issue [5,6]. Our limited knowledge of surgery in the older cohort is largely due to exclusion of older adults from clinical trials, a lack of trials designed specifically for older adults and the focus of trials (where available to older adults) on systemic therapy [5,6,7]. Even where high level evidence does exist, patient selection for treatment can be complex.

Due to often small numbers of trials primarily focused on older adults or lack of inclusion of older adults in larger trials, pooling of results for example through systematic reviews has been commonplace [8,9]. This approach is becoming outdated with the recognition and adaptations of trial design in recruitment of older adults and the publication of consensus guidelines from international organisations such as the International Society of Geriatric Oncology (SIOG). Using management of rectal cancer as one example, studies in large population-based datasets are now widely available [10] and there are expert recommendations, for example, from SIOG [11], and international consensus guidelines from numerous recognised organisations in this field [12].

Despite this, selection of patients for treatment remains an issue. One such example is in the field of breast cancer where there is potentially an equivalent treatment to surgery in older, frail patients—primary endocrine therapy (PET). Although PET may not be curative, it could control breast cancer for the remainder of an older adult’s life [13]. Despite there being a good evidence base for this, how to select patients for surgery or PET remains an issue. The most recent international guidelines published this year recommend that PET should only be given to patients with a life expectancy of <5 years [14], with previous guidelines limiting this to <2 years [15]; however, the 2020 report of the National Audit of Breast Cancer in Older Patients (NABCOP) in the UK found that around 10% of women aged 70–79 years and up to 47% of women >80 years did not have surgery [16], so there are clearly a number of other factors at play here. Interestingly, in the field of colorectal cancer, up to 74% of older patients stated they would refuse or be reluctant to receive treatment leading to severe functional impairment [17] with concerns regarding mortality, impairment of physical and mental function and the possibility of needing permanent residential care. There is clearly a discrepancy between recommendations and what is happening in clinical practice and reasons for this are multifactorial and complex and may include socio-economic status, comorbidity, geographical location of the patient, functional status and, perhaps most importantly, patient choice [18]. This creates individual challenges for surgeons who may be concerned with oncological outcome and geriatricians who may be concerned more with maintaining functional independence; however, the challenge to deliver patient-centred care is the focus of both specialties.

Therefore, current recommendations are difficult to interpret considering older adults with cancer as a whole and should be considered on an individual basis; they are not necessarily reflecting individual issues and preferences of older adults which do impact on treatment goals, decisions and cancer treatment outcome, such as social and behavioural changes over time [19,20]. Healthcare professionals have a duty to consider these issues when discussing surgical treatment options with older adults. Furthermore, trialists have a duty to consider these factors when designing clinical trials.

Reasons for the lack of inclusion of older adults in cancer clinical trials are multifactorial and challenging in themselves, but include factors relating to study design, patient factors and motivation of funding bodies and agencies [5]. Potential solutions include alternative study designs focused on the treatment goals of older adults, additional funding and resources to enable patients with multiple comorbidities including cognitive impairment, to participate and to raise awareness of the issues with trial sponsors and funding bodies [21,22]. Numerous published guidelines produced by overarching bodies do exist regarding suggestions for designing clinical trials in older adults with cancer; however, many recommendations remain challenging to implement in clinical practice and there is unlikely to be a ‘one size fits all’ approach given the diversity of the patient population we are discussing and geriatric oncology services available.

To give an example, the Japan Clinical Oncology Group (JCOG) has established a policy for geriatric cancer research [23] based on the current situation of geriatric patients with cancer in Japan. They have devised a set of suggestions for widening eligibility and exclusion criteria specific to research with older adults and suggest that restrictions based on comorbidities should only apply to those most severely affected by comorbidity. Furthermore, they suggest that clinical trials should not have a uniform maximum age requirement. To combat this, they suggest more frequent safety evaluations. Different study endpoints, such as physical and cognitive function, in addition to common endpoints, such as overall survival, should be considered. The JCOG guidance suggests that randomised controlled trials (RCTs) remain the gold standing when asking ‘which treatment is better’, but an observational study of a broader population is suitable for investigating actual conditions of older patients. These factors require a change in the mindset of treating surgeons and geriatricians and patients, which may be more difficult to achieve.

Many of these points are echoed by the American Society of Clinical Oncology (ASCO) recommendations designed to improve the evidence base in this area [24]. They have documented a number of action points for researchers to consider when designing trials such as providing rationale for restricted eligibility criteria and incentivising studies for including older adults. Furthermore, they highlight the input required from governing bodies, sponsors, funders and journals, both to commit to cancer research in older adults and also collaborate to develop common datasets in this age group. More pressure on governing bodies such as the European Medicine Agency and the US Food and Drug Administration is required in order to ensure adequate collection of data in older adults [25]. The challenge in achieving this requires widespread collaboration from overarching government and public health bodies, not solely at the level of the treating physician.

Trial design in itself is an issue. For example, RCTs of treatment versus non-treatment or comparing two different treatments, where the treatment(s) has potential side effects with little benefit, may be less attractive to older adults [5]. Alternative options, such as prospective cohort studies or retrospective evaluation of national population-based data sets, may answer questions regarding oncological outcomes based on what treatment the patient received [26], for example, in trials of de-escalation of surgical treatment. The Alliance for Clinical Trials in Oncology and the SIOG position article on this topic suggests that where RCTs are not feasible, large observational cohort studies or registries within the community should be established preferably in parallel to randomised trials so that treatment patterns across different settings can be compared with impact on outcome [25].

Overall endpoints of clinical trials need to be designed with the older population in mind. The Patient-Centered Outcomes Research Institute (PCORI) has invested a significant amount in patient-centred research that specifically targets the needs of older adults. They suggest that when comparing the benefit of two interventions in older adults the following should be considered: absolute risk difference, competing risks, life expectancy, the difference between chronological and physiological age, and patient preferences [27].

This topic of considerations of trial design in older adults with cancer has been summarised by Soto-Perez-De-Celis and Lichtman [28] who agree with expanding eligibility criteria, designing trials specifically for frail individuals, selecting more realistic endpoints and utilizing novel trial designs such as allowing patients to choose between treatments with their surgeons and geriatricians (rather than randomisation) as well as using components of geriatric assessment (GA) within clinical trials.

Designing the ‘ideal’ trial for older adults with cancer is a complex issue beyond the scope of this article; however, it is likely there is not a ‘one size fits all’ approach. Patients, physicians, researchers, trial funders/sponsors and governing bodies need to take a more flexible approach to clinical trials and be held accountable for inclusion of older adults in trials. This requires thoughts and inputs from surgeons and geriatricians together.

### 2.2. Difficulty in Selecting the Most Appropriate Surgical Procedure

Standard of care treatment can be multimodal including surgery, chemotherapy, radiotherapy and targeted therapies, as indicated by tumour type and stage; however, some reduction in cancer-directed therapies as a compromise in oncological outcome, in order to avoid some other risk or gain some small benefit (for example, symptom control in palliation) may be acceptable in the older population. The decision for or against surgery and the extent of that procedure, is complex and dependent on what options (surgical or non-surgical are available) as well as the physician and patient’s perception of fitness and frailty and individual preferences.

#### 2.2.1. Impact of Fitness and Frailty

Selecting a patient for less invasive surgical options or no surgical treatment for cancer, is primarily dependent on comorbidities; however, the impact of these comorbidities on a patient’s physiological function and likely impact on postoperative recovery is patient specific. The importance of preoperative assessment of fitness and frailty in the older adult with cancer has been extensively investigated and a number of guidelines written; however, again how to use this data in clinical practice remains less than straightforward.

A comprehensive systematic review by Huisman et al. [29] set out to determine which preoperative assessment tools (commonly used in GA) were most able to predict adverse postoperative outcomes. All domains were important; however, frailty seemed to be the most significant predictor.

Best Practices Guidelines from the American College of Surgeons National Surgical Quality Improvement Program (NSQIP) and the American Geriatric Society initially provided a resource for nine areas of preoperative assessment including cognitive/behavioural disorders, cardiac evaluation, pulmonary evaluation, functional/performance status, frailty, nutritional status, medication management, patient counselling and preoperative testing [30]. The second part of the guidance [31] targets the rest of the perioperative period, extending through the postoperative period to discharge. The guidance states that evaluation of the patient for frailty syndromes and documentation of their frailty score is recommended. Multiple definitions of frailty and how this should be measured were referenced in this paper.

A systematic review by Aucoin et al. [32] found frailty to be measured by 35 different instruments with different accuracy and feasibility. In summary, they found strong evidence in both areas to support the Clinical Frailty Scale; however, the Fried Phenotype is an alternative that requires a trade-off of greater accuracy with lower feasibility. A further review by Eamer et al. [33] risk assessed tools which could be utilised preoperatively to predict morbidity and mortality in older surgery patients. They again recognised different definitions and measurement of frailty. Overall, they felt that the most promise was found in the NSQIP preoperative mortality predictor, modified frailty index (MFI) and the Surgical Risk Preoperative Assessment System (SURPAS). Although frailty is clearly a significant issue which should be assessed preoperatively, how this should be achieved remains uncertain. In addition to frailty, other geriatric syndromes are important prognostic factors for postoperative complications with some potentially modifiable risk factors such as cognitive state, nutritional state and smoking status [34,35].

Preoperative cognitive function is becoming recognised as an important factor. A retrospective observational study of 251 older patients undergoing elective surgery for solid tumours by Hempenius et al. [36] observed that preoperative cognitive function and severity of surgical procedure were independent risk factors for postoperative delirium. This agrees with findings in a prospective study by Ristescu et al. [37], of 131 elective older adults with solid tumours, who found that preoperative cognitive impairment as well as renal dysfunction were associated with postoperative delirium.

Malnutrition is another key indicator of poor clinical outcomes for people with cancer [38] which can lead to a multitude of problems including decreasing efficacy of cancer therapy and reduction in quality of life [39]. A recent systematic review and meta-analysis on the subject [40] identified 42 studies which found that decreased food intake was associated with increased mortality from cancer treatment (both surgery and other treatments).

In the Bridging the Age Gap (BTAG) in Breast Cancer UK study of 3375 older women with breast cancer; age, frailty, dementia and a number of comorbidities were predictors of no axillary surgery [41]. With regards to post-treatment quality of life and functional independence, patients receiving either surgery or PET, both exhibited a decline in global health status scores; however, the decline was sharper in the surgery group. Moreover, this score failed to return to baseline level.

All of the above quoted studies suggest that these factors (cognitive impairment, frailty, comorbidity) should be routinely screened prior to cancer treatment in older adults to help in the decision-making process regarding treatment. An example is the Age Gap Decision Tool generated by the BTAG study group (https://agegap.shef.ac.uk (accessed on 27 January 2022)) which predicts survival in older women with breast cancer at diagnosis. The tool considers simple measures of frailty and comorbidity and has been validated for use in patients with oestrogen receptor-positive breast cancer [42].

#### 2.2.2. Extent of Surgical Procedure

In addition to measured frailty and comorbidity, extent and duration of surgical procedure may have an impact on post-operative outcome in older adults with cancer in terms of immediate recovery, functional recovery and oncological outcomes. The extent of surgery may be minimally-invasive compared to an open approach, but it could also mean a decision between more extensive dissection in abdominal surgery or between reconstruction or no reconstruction in breast surgery, for example.

A limited approach versus traditional open procedures has been shown to be beneficial across many cancer types. Laparoscopic versus open cancer surgery for older adults with colorectal cancer has better short-term outcomes in terms of recovery and length of hospital stay [43] and comparable oncological outcomes up to at least 5 years [44] with similar findings in gastric cancer surgery [45]. A thoracoscopic approach for lung cancer surgery, compared to open, not only contributes to reduced surgical trauma and preservation of chest wall mechanics, but reduces postoperative morbidity, mortality, delirium and lower narcotic requirements [46]. Although these recommendations do apply to all patients irrespective of age, it is especially relevant to discuss these factors when planning surgical treatment in the older cohort, for example, when discussing reconstructive procedures in breast cancer surgery (often requiring longer operative duration and greater length of hospital stay), or stoma formation in colorectal cancer resection (to negate the risk of anastomotic leak after primary anastomosis).

Using the example of gastric cancer [47], it is noted that despite generally having more lymph node involvement, older patients often undergo more limited lymphadenectomy with little impact on overall survival. Impact on overall survival in real terms should be discussed with the patient; more extensive surgery with little or no impact on overall survival may not be deemed suitable by the patient, even at the expense of local control of disease, for example.

The extent of surgery or magnitude of a specific surgical intervention is hard to quantify and differs from person to person. How to integrate this into existing pre-operative assessment measures, manage patient expectation, preferences and values to make informed treatment decisions is a challenge. There are some studies looking at ways to attempt to quantify the extent of a surgical intervention which may be used to direct treatment decision making. Schwarze et al. [48] ran a retrospective cohort study and modified Delphi procedure in an attempt to develop a list of high-risk operations (not specifically for cancer) in older adults. They looked at over 4 million admissions of patients ≥65 years of which over 2.5 million had a procedure. Modified Delphi procedure consensus of a panel of surgeons and proportion agreement in the Nationwide Inpatient Sample was used to define high-risk operations and a list of procedure codes has been developed following this. In general, high-risk procedures performed on patients ≥65 years of age had double the mortality compared to patients <65 years.

A cohort study of over 400,000 patients by Shinall et al. [49] aimed to assess if frailty was associated with increased post-operative mortality. This study was not specifically focused on older adults or adults with cancer; however, even minor surgical procedures were associated with higher risks for patients with frailty. They concluded that surgeons should consider whether the potential benefits of surgery warrant the increased risk in any frail adult. This returns us again to the challenge of how to measure frailty.

Preoperative assessment can aid in care coordination and provide specific targets for intervention and should include assessment of frailty; however, there remain many methods on how to do this. How we choose the right patient for the right treatment, surgical or non-surgical, should be based on an individualised personalised approach; however, comorbidity should not be seen as a barrier to surgery, but a hurdle to overcome. With this in mind it is important to think about how we can best optimise these patients for surgery.

### 2.3. How to Optimise the Individual Older Adult for Surgery

In this section we will talk about the function of prehabilitation and benefit in cancer surgery; the potential role of GA or a screening tool to identify which surgical candidates may benefit from preoperative optimisation; and the use of enhanced recovery programmes in the postoperative setting.

#### 2.3.1. Prehabilitation

Prehabilitation describes multimodal, needs-based interventions designed to improve the physiological, metabolic and psychological resilience of an individual prior to an expected major stressor, such as surgery [50]. Much like the well-established rehabilitation programmes, prehabilitation and managing functional status requires input from multiple team members including but not limited to: physiotherapists, occupational therapists, pharmacists and psychologists [51]. Exercise training before elective adult major surgery is feasible and safe; however, clinical effectiveness remains uncertain [50]. There are few published clinical trials of prehabilitation in older adults undergoing cancer surgery; however, the benefit of successful intervention cannot be predicted.

The Geriatric Oncology Surgical Assessment and Functional rEcovery after Surgery (GOSAFE) study [52] is a multicentre international prospective cohort study which collected data on 1005 patients aged ≥70 years before major elective surgery. A plethora of information was collected around frailty and functional recovery before and after surgery, looking for predictors of good quality of life (QOL) and functional recovery at 6 months postoperatively. Preoperative frailty predicted 3 and 6 month morbidity and mortality, reduction in quality of life and decline in functional recovery [53]. Thereby, by taking steps to improve preoperative frailty, we can hypothesise that these outcomes will be improved.

A systematic review by Daniels et al. [54] assessed prehabilitation in preparation for abdominal cancer surgery. In total 33 studies were included covering the following interventions: exercise, nutrition, psychological input, comprehensive geriatric assessment (CGA) and optimisation, smoking cessation and a combination of interventions. Conclusions were limited by the quality of the included studies but exercise, nutritional and multimodal prehabilitation seemed to reduce morbidity after abdominal surgery; however, data specific to older patients was sparse which was also a problem in an earlier review by Bruns et al. [55].

Specific to the older population, Li et al. enrolled 42 older adults undergoing elective cancer resections, to a prehabilitation programme consisting of an exercise programme, nutritional evaluation and anxiety reduction postoperative functional recovery with 45 adults prior to introduction of the programme. The prehabilitation group had improved postoperative functional recovery at one month and reported higher levels of physical activity then before surgery [56].

A number of hopefully pivotal studies on prehabilitation are ongoing. The PROADAPT study led by Roche et al. in France [57] is a prospective pilot study conducting both CGA and prehabilitation in 122 older adults planned for curative treatment. The PREHAB study based in Canada led by McIsaac et al. [58] will randomise older patients having elective intra-abdominal or intra-thoracic cancer surgery to home-based exercises prehabilitation versus standard care.

Prehabilitation has shown some promise in older adults undergoing surgery but raises many of the same problems with implementation as with GA—it could be time consuming and relies on additional resources.

#### 2.3.2. Geriatric Assessment

A map of the current services and projects in the UK in the field of geriatric oncology was performed by Gomes et al. in 2020 [59]. It concluded that although the care of cancer patients was a significant part of daily practice, routine care of these patients did not include a formal geriatric or frailty assessment/management and the use of treatment toxicity prediction tools was not standard practice. The models of care were very heterogeneous and adapted to local priorities.

The benefits of CGA have been shown in other areas of geriatric medicine such as stroke medicine [60] and help to reduce mortality, maintain physical function and reduce the likelihood of nursing home admission [61]. In up to 70% of older patients, CGA can reveal problems otherwise not identified through a traditional oncological assessment [62].

The most recent SIOG guidelines on the subject recommend use of a GA in all older adults with cancer which should include assessment of: functional status, comorbidity, cognition, mental health status, fatigue, social status and support, nutrition and presence of geriatric syndromes [15]. This is reiterated in latest guidelines from the National Comprehensive Cancer Network, which provides comprehensive guidelines for components of GA [63].

An established CGA tool for use in cancer patients in general has been developed and tested in the USA by Hurria et al. in 2005 [64]. This tool is unique and important in that it was the first to be specifically designed and validated for use in oncology patients, as opposed to older adults in general. The measures used evaluated all of the various domains of CGA and were selected for their reliability, validity, brevity and prognostic ability to determine risk for morbidity and mortality in an older patient. Hurria’s tool, however, was validated in cancer patients undergoing chemotherapy.

Since this time, there has been a plethora of work on the use of GA to guide decisions and intervention for cancer, with the results of recent trials presented at the American Society of Clinical Oncology (ASCO) conference. In 2020, [65] three of the four RCTs presented were focused on patients with solid tumours commencing systemic therapy. The fourth trial by Qian et al. [66] investigated patients undergoing surgery for gastrointestinal cancers. Qian et al. [66] randomised older adults planning to undergo surgery for gastrointestinal cancers to receive a perioperative geriatric intervention, or standard care. There were no differences between groups and post-operative length of stay, admission to intensive care and readmission rates; however, the intervention group reported lower postoperative symptoms (as measured by the Edmonton Symptom Assessment System) and less symptoms of depression.

Moving to ASCO 2021, the focus of studies reporting GA remained centred on systemic therapy [67,68,69], as well as identifying potential barriers of GA and how to address these [70]. Serna et al. [71] performed a retrospective analysis of two consecutively-treated cohorts of older patients with head and neck squamous cell carcinoma (HNSCC), one cohort treated based on CGA and one as a control. Patients were more likely to receive standard treatment (compared to adjusted, palliative or best supportive care) in the CGA cohort; however, treatment completion rate and overall response rate remained the same.

A number of other notable studies in this field have found mixed results.

A cross-sectional study by Sourdet at al [72] implemented CGA prior to cancer treatment for 418 older patients with solid or haematologic cancers. Initial cancer treatment plan was changed in 16.7% of patients and was associated with cognition, malnutrition and low physical performance.

Ommundsen et al. [73] randomised 172 older patients with colorectal cancer to either preoperative GA followed by a tailored intervention or standard care and found no statistical difference between either group and rate of complications, reoperations, readmission or mortality in frail older patients. Mohile et al. [74] randomised 541 participants with solid tumours or lymphoma to receive either a tailored GA with recommendations for or standard care and found that including GA in the clinic increased patient and caregiver satisfaction; however, quality of life outcomes did not differ. Ørum et al. [75] recruited 363 older adults with head and neck, lung, upper gastrointestinal or colorectal cancer to complete CGA and be randomly assigned to either a control group with no follow-up or intervention group with tailored follow-up. In frail and vulnerable patients, no differences in ability to complete treatment planned, activities of daily living, physical performance or hospitalisation were found.

Despite the evidence and recommendations for GA already published, the ability of GA to influence cancer treatment decisions and outcomes in cancer patients has yet to be firmly established and a number of challenges are yet to be resolved including the time-consuming nature of GA and lack of personnel/resources to implement. The concept of a screening tool to determine who should receive full GA has been investigated. Decoster et al. on behalf of SIOG [76] reviewed 44 studies reporting on the use of 17 differing screening tools. The tools most studied are G8, Flemish version of the Triage Risk Screening Tool (fTRST) and Vulnerable Elders Survey-13 (VES-13). Different tools demonstrated associations with different outcome measures. They concluded that screening tools do not replace GA but are recommended in a busy practice to identify those who might most benefit from full GA. A review by Garcia et al. on the subject [77] reviewed 17 studies of 12 tools and recommended G8 or VES-13 to screen for potential issues in older adults with cancer.

Most of the evidence on the benefits of GA and intervention are from studies looking at cancer in general, with few specifically focused on surgery; however, similar observations in cancer surgery are starting to emerge. There are a few ongoing randomised studies globally exploring the use of CGA in surgical cancer patients [78,79] and the growing body of evidence should help to answer the remaining questions surrounding utilisation and implementation of CGA in clinical practice.

#### 2.3.3. Enhanced Recovery

There is a wealth of evidence in the literature in support of enhanced recovery protocols; however, yet again little focus has been on the older adult.

The ERAS protocol (Enhanced Recovery After Surgery) is a multimodal pathway aimed to reduce surgical stress and allow rapid postoperative surgery. ERAS has widely been adopted in some cancer centres, but again its specific role in the older adult is lacking. The benefits of ERAS in the wider population have been proven and include shorter postoperative recovery time, reduced post-operative complications as well as being cost-effective [80]. The protocol has been widely adopted internationally initially in colorectal [81], later in gastrointestinal [82] and more recently gynaecological cancers [83].

Although not widely adopted, there may be merit in introducing ERAS to other cancer types, even those historically considered less invasive, such as breast surgery. An ERAS pathway for total mastectomy has been shown to reduce use of analgesia and antiemetics following surgery and promote successful early recovery [84].

The slow uptake of ERAS in other cancer types, is presumed partly due to the mindset of the clinical team and their challenge of traditional surgical practices [85]; however, with growing evidence in this field and normalisation of such protocols, ERAS may become standard of care in all oncological practices in the future, with a special focus on the older adult.

The American College of Surgeons (ACS) Geriatric Surgery Verification (GSV) Programme presents 32 new surgical standards specifically designed to improve surgical care and outcomes for older adults. The standards provide a framework for a team approach of specialists from different disciplines, to continuously optimise care in this cohort and include guidance on pre- and postoperative management, importance of overall health goals, community outreach and education [86].

## 3. Moving Forward and Collaborative Working

Many of the topics discussed in this article require comprehensive papers in their own right; however, one element they all have in common is the need for the surgical oncologist and geriatrician to work together.

Over the last decade the benefits of integrating a geriatrician into surgical practice has been noted; however, implementing this is clinical practice is fraught with challenges including time constraints of individual job plans, availability of funding and enthusiasm of colleagues. A potential alternative may be to use GA as a surrogate for an in-person geriatrician, but lends to a separate list of challenges, again including limitations of time and resources, as well as deciding which of the multiple GA tools in existence to utilise and how best to act upon the results.

The benefit of an integrated surgical and geriatric approach has been a historical success in the field of orthogeriatrics, particularly in relation to hip fractures where a combined orthogeriatric approach has been proven to reduced average length of hospital stay, increased rate of discharge own home (compared to care home) and improved management of coexisting medical comorbidities [87].

Using breast cancer surgery as an example, the most recent joint guidelines from SIOG and European Society of Breast Cancer Specialists (EUSOMA) published in July 2021 [14] have made a number of significant recommendations which will impact the running of a surgical service. These include routinely screening for frailty in all patients aged ≥70 years at presentation with cancer and applying a screening tool as a minimum starting point (for GA) prior to any treatment decision making. Integration of these recommendations globally is a real challenge for the decades to come.

There are some studies investigating how the collaboration between surgeons and geriatricians can be achieved. The majority of these are focused on surgical procedures as a whole and not specifically for cancer surgery; however, the same principles apply and can be considered in practice.

A survey of geriatricians in Australia and New Zealand [88] identified a number of barriers to an integrated service including the lack of funding for staffing, encroaching on existing services and competing clinical priorities. The key barrier at the healthcare professional level was the lack of clarity of roles within the perioperative team. They suggest future work in this field to include application of patient-reported measures and qualitative research with patients to inform patient-centred perioperative care. Similar work in the UK within surgeons and geriatricians group and suggested variables such as ownership and location of the patient and education as key variables [89]. The main obstacle preventing integrated working was the concern of de-skilling the surgeons, narrowing their role to ‘technician’. There are significant human factors here to overcome. Several models of care were suggested: 1. Surgeons manage patients on surgical wards with input on request from other physicians including geriatricians; 2. An expanded role for surgeons, trained to manage medical problems in complex older patients, geriatricians have an advisory and teaching role; 3. Joint care on a surgical ward between surgeons and geriatricians; and 4. Transfer of patient from surgical to medical ward and from surgical to geriatrician led care postoperatively, the two specialties remain separate. Problems with implementing any of these methods which are a change to usual standard of practice are the lack of recognition that a change is required and the lack of evidence for these models in practice.

Shipway et al. [90] implemented a study at a single centre in London looking at a geriatric surgical liaison service for emergency and elective gastrointestinal surgery. The intervention included open access referral for CGA, twice weekly ward rounds on selected patients led by a consultant geriatrician accompanied by members of the surgical team, twice weekly discharge planning meetings involving multiple team members and access to a geriatrician-led surgical rehabilitation ward. The process was associated with a mean reduction in length of stay of 3.1 days for all surgical patients aged >60 years.

Vilches-Moraga et al. [91] present options for assessment and patient centred interventions between geriatricians and older emergency general surgical patients in Salford. Options considered include: (1) Single organ speciality physicians; (2) general physicians/surgeons/anaesthetists sharing care; (3) perioperative specialists; and (4) geriatrician led cross-speciality team. The system they have implemented (Salford-perioperative care of older people—general surgery POPS-GS) consists of a general surgical in-reach service. Two consultant geriatricians provide five direct clinical care sessions in the general surgical wards weekly and the service has shown decreased length of stay by 3.2 days.

Magnuson et al. [92] suggest further models of care such as geriatricians conducting GA in an outpatient setting, a specific clinic led by oncologists or by embedding a geriatrician into the clinic. They recognise the challenge with staffing these services and suggest utilising geriatrics-trained nurse practitioners or physicians’ assistants. Furthermore, they suggest that trained geriatric oncologists become the primary care providers for older adults with cancer, instead of the surgical oncologist or general geriatricians.

A final example by Presley et al. [93] describes the Cancer and Aging Resiliency (CARE) clinic set up in a single institution in the US. This is a consultative model in which patients are seen for a ‘one-time’ visit where geriatric deficits are assessed and interventions prescribed at this visit. Ongoing oncological needs are fulfilled separately through the primary oncology care provider.

Many of these published examples are descriptive in nature and long-term outcomes are yet to be fully realised; however, these studies give promise to the potential options for expanding a geriatric oncology service in various institutions, with varying degrees of input from surgeons and geriatricians. Clearly the most appropriate model will vary from institution to institution. Use of a geriatric oncology nurse, clinical nurse specialist or advanced clinical practitioner in geriatrics, rather than the direct involvement of a geriatrician, may be more suitable in some centres [94,95]. This could potentially mean that GA is performed in the absence of a physical geriatrician, but their presence remains important in guiding intervention based on GA outcome.

**Figure 1:**
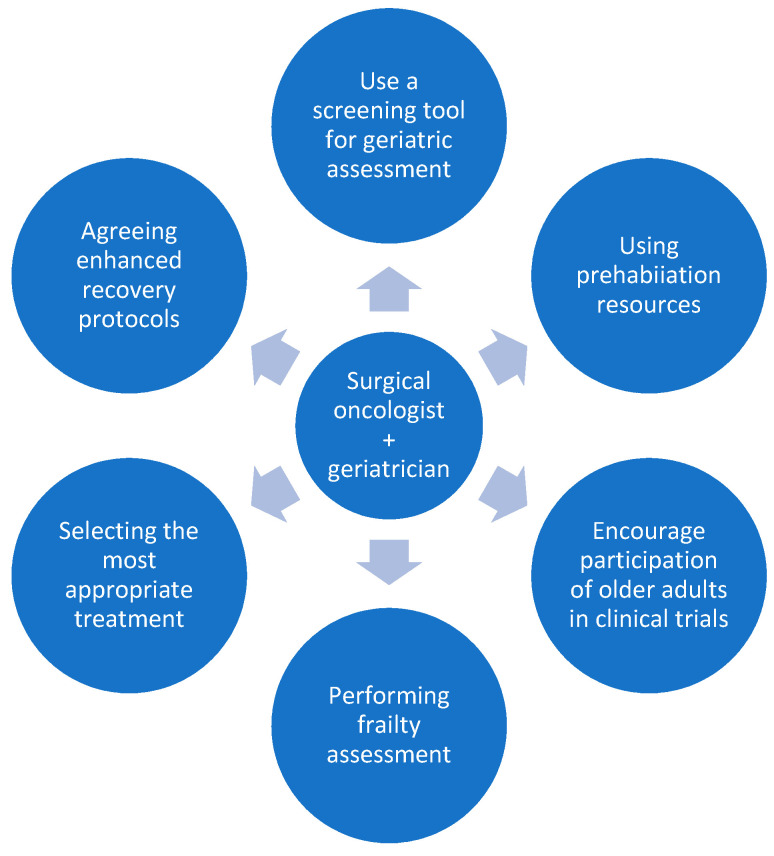
The potential roles of the collaborating surgeon and geriatrician.

Figure 1 summarises the potential roles of the collaborating surgical oncologist and geriatrician.

There are a number of key themes that have reoccurred throughout this article. These include recognising that the treatment goals of older patients may be different compared to their younger counterparts and utilising the concept of personalised medicine in each individual patient. A good working relationship between surgical oncologists and geriatricians is vital in moving forward and a combined approach will help to maximally address the challenges in the management of older adults with cancer. This collaboration should focus on increasing the evidence base for surgical options for older adults with cancer, working together to decide the most appropriate surgical treatment for each individual patient and optimising the patient for this. How this is done in practice will differ from centre to centre and depend on resources available at individual institutions, but one factor will be constant irrespective of location—the surgical oncologist and the geriatrician must work together as one united team.

## Figures and Tables

**Table 1:** A summary of the challenges and potential solutions facing clinicians treating older adults with cancer.

Challenge	Potential Solutions
Differing evidence base for surgery in older adults compared to younger	- Better recruitment of older adults to clinical trials - Consideration of the different treatment goals in older compared to younger adults (maintenance of quality of life and functional recovery compared to curative intent) - Moderation in trial design for older adults
Difficulty in selecting the most appropriate surgical procedure	- Individual assessment of fitness and frailty - Consideration of extent of surgical procedure and objective assessment of this - Evaluation of the impact of treatment on each patient as an individual
How to optimise the individual older adult for surgery	- Evolving evidence shows that prehabilitation may be able to minimise postoperative decline - Consider use of geriatric assessment as routine in surgical practice to identify which older adults may be at risk of postoperative deterioration - Use of enhanced recovery after surgery protocols for all oncological diagnoses
Collaboration between surgical oncologist and geriatrician	- Recognise there is a problem which requires collaboration - Novel methods for introduction of geriatrics into surgical practice - Utilise resources/personnel dependent on services available
